# Prescriptions and Dispensing

**Published:** 1908-02-22

**Authors:** 


### Therapeutics.

### PRESCRIPTIONS AND DISPENSING.

Turpentine may be emulsified by means of gum acacia, 1 drachm of the powder being taken for each 2 fluid drachms of the turpentine. This emulsion can be better made in a bottle than in a mortar, in the following manner: Take a dry bottle and put into it the powdered gum, and then the turpentine. Shake well. Then add, in one quantity, an amount of water equal to that of the turpentine employed, and again shake well. Add more water, but only half as much this time as before; repeat the shaking, and add more water in quantities equal to the last, shaking after each addition.

The use of gum acacia in making up resinous tinctures in an aqueous vehicle has been referred to already; tincture of cannabis indica, simple or compound tincture of benzoin, and others, may be treated in the manner described for tincture of tolu. Balsam of peru is treated as an essential oil.

Gum tragacanth, though generally much inferior to gum acacia as an emulsifying agent, is useful in certain cases, usually as an aid to some other agent. We shall refer to it in what follows in connection with some of the other substances dealt with.

It is interesting to notice that the most perfect emulsion in nature—namely, milk—contains no gum. Milk consists of about 88 per cent. of water, with fat, lactose, casein, and mineral salts. The fat is in the form of extremely fine globules, much smaller than those produced in most artificial emulsions; and although when milk is left at rest a great deal of the fat rises to the surface in the form of cream, the globules do not coalesce. The important agent in keeping the fat emulsified is the casein, and this constituent can be extracted from milk and used in other mixtures.

Casein is, of course, a protein; in extracting it from milk it undergoes a certain amount of change. The unaltered substance as it exists in fresh milk is more correctly termed caseinogen, though it is often termed casein. The extracted casein is not soluble in plain water, as caseinogen is; but if treated with a very little alkali and water the greater part dissolves, the remainder being suspended, and giving a milky appearance. The mineral salts of the milk, particularly the phosphates, assist considerably in keeping "casein" in solution. Casein is now prepared from milk on a very large scale, and it is used commercially for a great variety of purposes. A "soluble casein" may be prepared as follows:—

| | | |
|---|---|---|
| ℞ | Commercial Casein (in fine powder) | 8½ parts |
| | Sodium Bicarbonate ... ... ... | 1½ parts |

When this mixture is treated with water, combination occurs, and sodium caseate is formed; the dry powder swells and becomes gelatinous; if sufficient water is employed the greater part dissolves. Such a "soluble casein" will go farther than an equal weight of gum in making an emulsion, as in the following example:

| | | |
|---|---|---|
| ℞ | Olei Ricini ... ... ... ... ... | ℥ijss. |
| | Sodii Caseatis ... ... ... ... | ʒiij. |
| | Aquam Chloroformi ... ... | ad ℥xvj. |

The soluble casein should be rubbed up with enough water to produce a moderately thin paste, after one or two minutes have been allowed for the swelling of the material that occurs; the oil may then be added in about four portions, with thorough mixing between each addition, and alternation with small quantities of the chloroform water to keep the consistence right; the rest of the water may then be added gradually to make up the 16 oz. A beautiful milky emulsion is obtained. On standing, however, it tends to separate into two layers, the upper resembling cream, with nearly clear fluid beneath it. The oil globules do not coalesce, however, so that the emulsion may be re-made at once with a very little shaking. When any casein emulsion has to be kept for any length of time, chloroform water, or an alcoholic preparation, or some other innocuous preservative, should be included in the prescription, because casein solutions are very favourable media for the growth of micro-organisms, so that they go bad more rapidly than acacia mucilage does, with the production of a most offensive smell.

Casein is not the only member of the protein class that is of use in making emulsions. The white of an egg can be used as an emulsifying agent. Preference is usually given, however, to the yolk. Egg-yolk is a remarkably efficient emulsifier, one yolk with a little gum tragacanth being sufficient to emulsify 5 fluid ounces of cod-liver oil. The following prescription may be taken as an example of such emulsions:

| | | |
|---|---|---|
| ℞ | Olei Morrhuæ ... ... ... ... | ℥v. |
| | Ovi Vitellum ... ... ... ... ... | j. |
| | Pulveris Tragacanthæ ... ... ... | gr. v. |
| | Elixir Saccharini ... ... ... ... | ʒss. |
| | Aquam Cinnamomi ... ... | ad ℥x. |

To dispense this, break the egg and carefully separate the white from the yolk as completely as possible; failure to remove all the white is sometimes a cause of trouble. Mix 2 drachms or so of the cod-liver oil with the gum tragacanth in a mortar, add the yolk, and triturate thoroughly; then add further quantities of oil, alternating sometimes with a little of the cinnamon water to keep the mixture of a good consistence. After all the oil has been incorporated, add most of the remaining cinnamon water in portions, retaining a little to dilute the elixir, which should be added last.

Yolk of egg is a useful means of emulsifying turpentine; and in this case, as in the acacia turpentine emulsion previously described, the operation is best carried out in a bottle. The yolk is first mixed in a mortar with twice its volume of water, and after straining through muslin, if necessary, 1 to 1½ parts of this mixture are taken for each part of turpentine. The diluted yolk is put into the bottle and shaken up; then the turpentine added all in one quantity, and the two well shaken together; the water is then gradually added, with more shaking. Egg emulsions, like those of casein, should contain some innocuous preservative.

*(To be continued.)*

